# Results of a patient survey using an online questionnaire after implant removal for breast implant illness

**DOI:** 10.3205/iprs000186

**Published:** 2024-03-11

**Authors:** Ursula Tanzella, Klaus Ueberreiter, Lola Fanny Krapohl, Armin Bell, Björn Dirk Krapohl

**Affiliations:** 1 Park-Klinik Birkenwerder, Birkenwerder, Germany; 2 University of Leipzig, Faculty of Medicine, Leipzig, Germany; 3 Department of Dento-maxillofacial, Reconstructive, and Plastic Surgery, Carl-Thiem Klinikum, Cottbus, Germany

**Keywords:** breast implant illness, silicone implants, capsular fibrosis

## Abstract

The use of silicone breast implants has a history of over 60 years. In recent years, specific health issues among implant wearers have repeatedly come into focus. The term “breast implant illness” has been circulating in scientific literature and on social media for several years. It describes a cluster of up to 60 different symptoms. The present results of an online survey conducted within a clinic’s patient population of the last 8 years show, among other things, the evolution of 8 reported symptoms before and after breast implant removal. In the comparison before and after, there is a significant reduction in the intensity of symptoms after implant removal. A causal relationship with the removal of the implants is to be presumed.

## Abstract (de)

Die Verwendung von Silikon-Brustimplantaten blickt auf eine über 60-jährige Geschichte zurück. In den vergangenen Jahren rückten immer wieder spezifische gesundheitliche Probleme bei den Implantatträgerinnen in den Fokus. Seit einigen Jahren kursiert der Begriff Breast Implant Illness in der wissenschaftlichen Literatur und auf Social Media. Dabei handelt es sich um die Beschreibung eines Clusters von bis zu 60 verschiedenen Beschwerden. Die vorliegenden Ergebnisse einer Online-Befragung innerhalb des Patientenguts der letzten 8 Jahre einer Klinik zeigen unter anderem die Entwicklung von 8 erfragten Beschwerden vor und nach Brustimplantatentnahme. Es zeigt sich im Vorher-Nachher-Vergleich eine deutliche Reduzierung der Beschwerdeintensitäten nach Implantatentnahme. Ein kausaler Zusammenhang zur Entnahme der Implantate ist zu vermuten.

## Introduction

Silicone implants have been used worldwide with increasing popularity since the 1960s. In the 21^st^ century, breast augmentation with implants consistently ranks in the top three of popular and requested procedures in annual statistics published by respective medical societies.

In the past, the body’s reaction to silicone in tissue has been a recurring focus. Two phenomena initially took center stage: capsular contracture and the potential association of symptoms from autoimmune or rheumatic conditions in silicone implant recipients. The former led to modifications in the implant surface to minimize the risk of severe capsular contracture, achieved through a textured coating. This textured surface significantly increases the silicone contact area compared to smooth implants.

The latter set of symptoms led, for example, in the U.S. to a period from 1992 to 2006 during which only saline-filled implants were approved. Since 2006, silicone-filled implants have been reintroduced in the U.S.

In recent years, there has been increasing reporting of a new complex of symptoms seemingly associated with breast implant recipients [1], [2]. This is not a defined disease but rather a cluster of up to 60 diverse symptoms. Some of these can be attributed to the autoimmune syndrome induced by adjuvants (ASIA), chronic fatigue syndrome (CFS), or connective tissue diseases such as collagen disorders, lupus erythematosus, scleroderma, and Sjögren’s syndrome [3], [4], [5], [6]. Additionally, there are overlaps with menopausal symptoms and psychiatric conditions, such as depression.

Due to the lack of a concrete entity for this complex of symptoms, it has been subsumed under the term “breast implant illness” and circulates not only in scientific literature but also, and especially, in social media channels. Currently, there are over 100,000 posts under #breastimplantillness on Instagram, indicating the significance of social media as a primary source of information for patients with ambiguous symptoms [7], [8].

To fill this information and consultation gap, it is crucial to raise awareness of this phenomenon among treating physicians.

## Material and methods

During the period from January 2016 to February 2024, a total of 225 patients with symptoms of breast implant illness were treated in our clinic, Park-Klinik Birkenwerder, Germany. The surgical approach is briefly explained as follows:

All patients had their existing implants removed. As a standard procedure, patients were offered volume replacement, which involved autologous fat transplantation using water-assisted liposuction according to the Beauli™ method after the removal of implants [9], [10], [11].

Within the existing patient collective, three different surgical options were performed in relation to implant removal:

- Pure implant removal without additional volume replacement.
- Implant removal and volume replacement through autologous fat transplantation using the Beauli™ method.
- Implant removal and simultaneous breast lift, either in combination with additional volume replacement using the Beauli™ method or without additional volume replacement.

For some patients, the implant removal was en-bloc, meaning it included the body’s own capsule without opening it. For all other patients, partial resection of the capsule was performed. All removed capsule components were sent for histopathological evaluation.

Among the patients followed up, one case of ALCL (anaplastic large cell lymphoma) occurred within the implant capsule.

Follow-up of symptoms using an online questionnaire: All patients were sent a Google Drive online questionnaire at least three months and at most one year after implant removal, which could be answered online. The responses were anonymized, ensuring no conclusions could be drawn about the sender. All patients had previously agreed to be contacted and surveyed.

**Figure 1:**
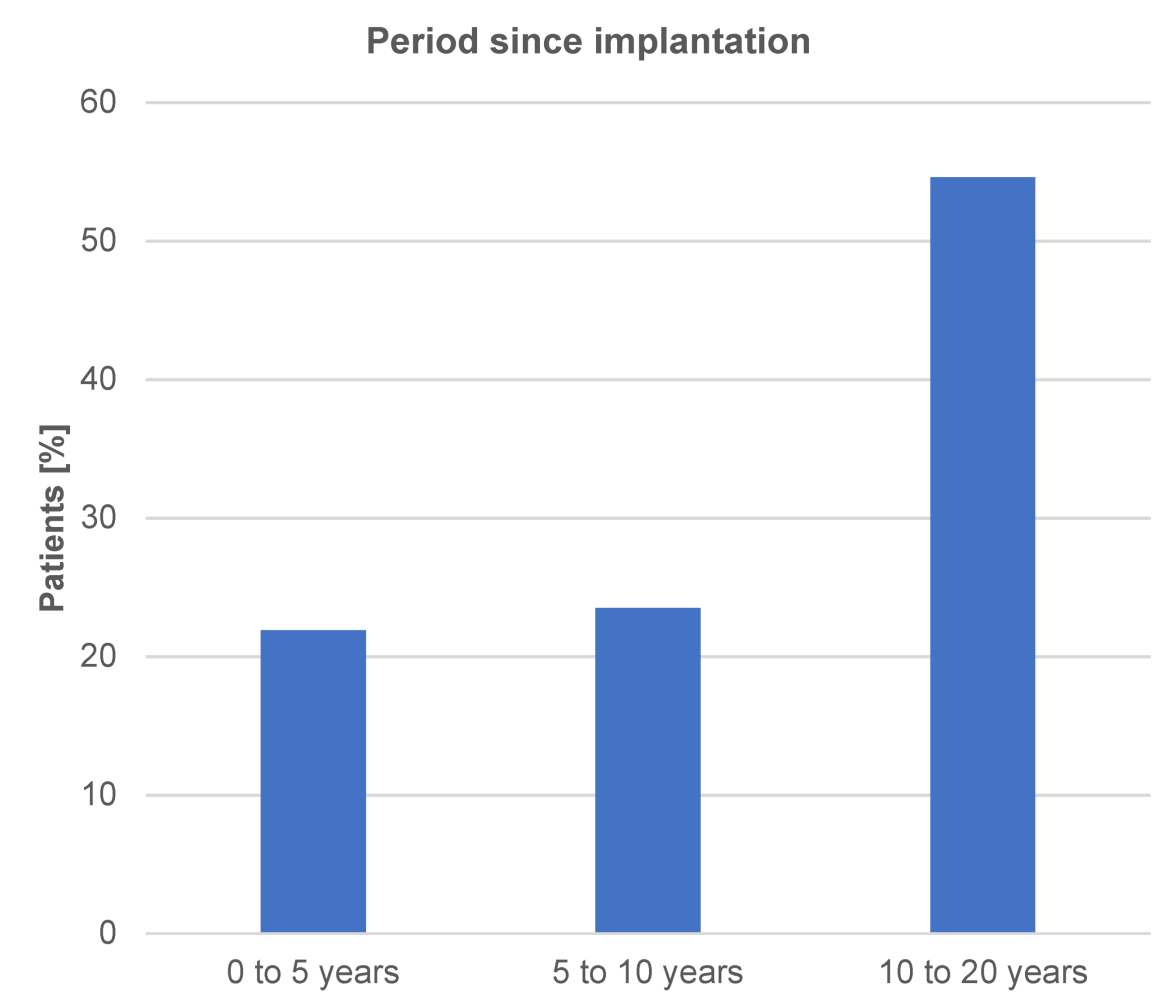
Time elapsed since implantation

**Figure 2:**
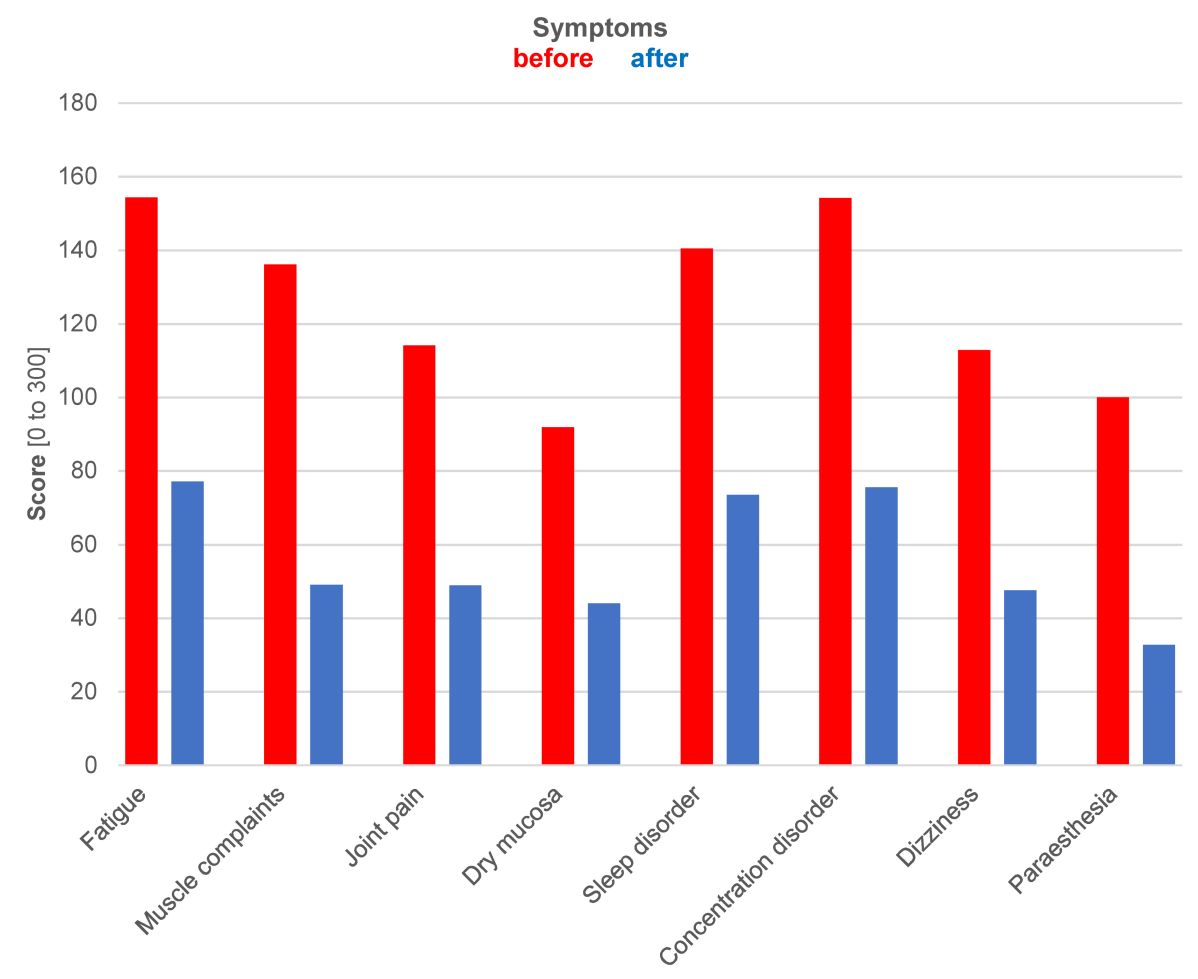
Development of symptoms in comparison: preoperative (in red) and postoperative (in blue), i.e., before and after implant removal

A total of 103 response forms were received, corresponding to a response rate of 45.8%. The questionnaire consisted of 19 questions and a free-text field. It covered the duration of wearing the implants (Figure 1 (Fig. 1)) and the intensities of the most common breast implant illness symptoms such as chronic fatigue, muscle pain, joint pain, dry mucous membranes, sleep disturbances, concentration difficulties, dizziness or weakness, tingling sensations, and numbness in the extremities before and at least three or a maximum of twelve months after implant removal. Four intensity levels per symptom before and after could be indicated (Figure 2 (Fig. 2)). The four intensity levels were validated with a score for each symptom to more easily visualize them in the before-and-after comparison: no symptoms – score value 0, mild symptoms/intensity – score value 1, moderate symptoms/intensity – score value 2, severe symptoms/intensity – score value 3. The score values were added in each symptom group, and a pre-to-post-operative comparison was later conducted.

**Figure 3:**
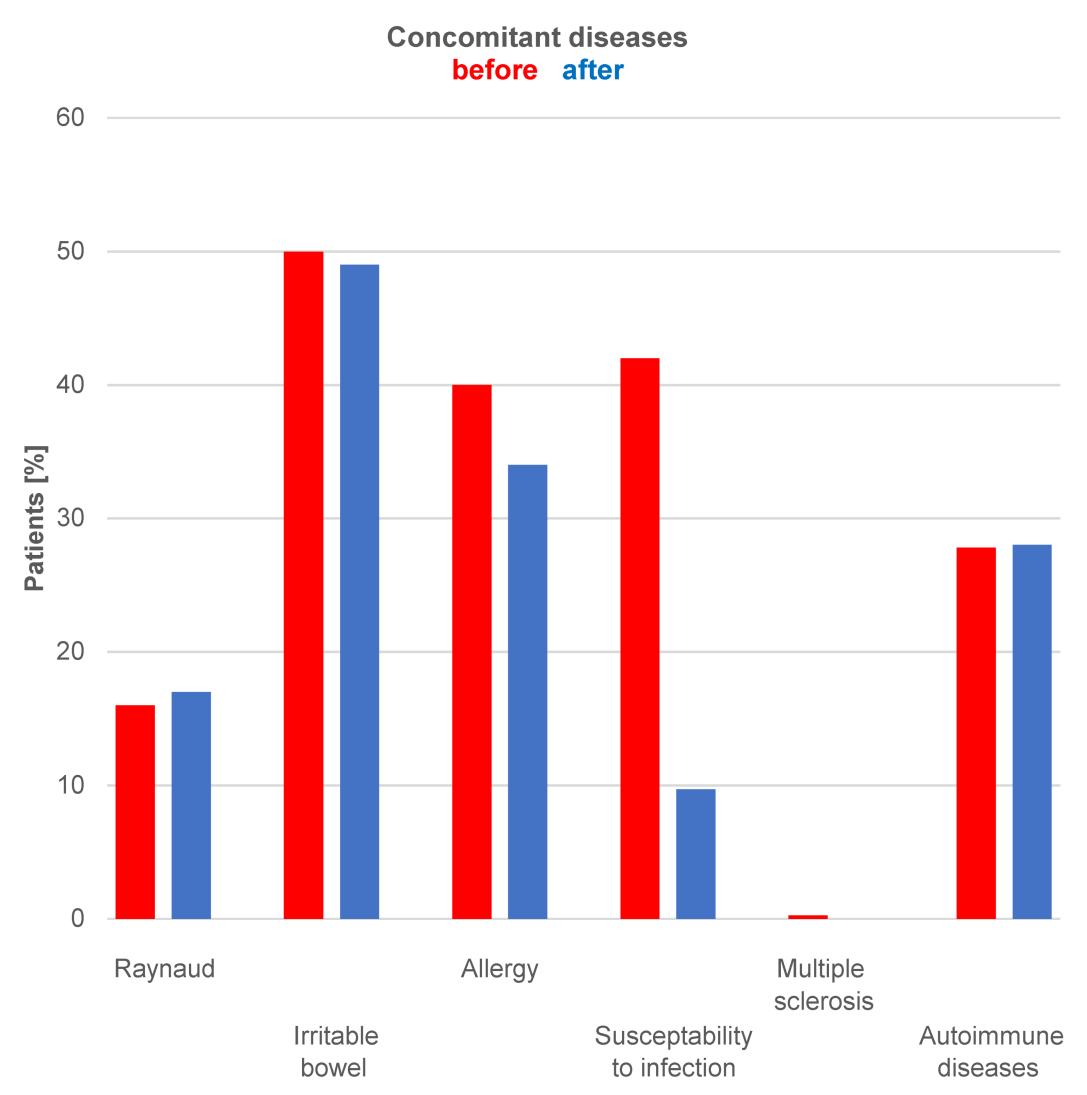
Development of comorbidities in comparison: preoperative (in red) and postoperative (in blue), i.e., before and after implant removal

Additionally, the occurrence of comorbidities such as Raynaud’s syndrome, irritable bowel syndrome, allergies, susceptibility to infections, multiple sclerosis, and autoimmune diseases were queried and numerically summarized in the before-and-after comparison (Figure 3 (Fig. 3)).

**Figure 4:**
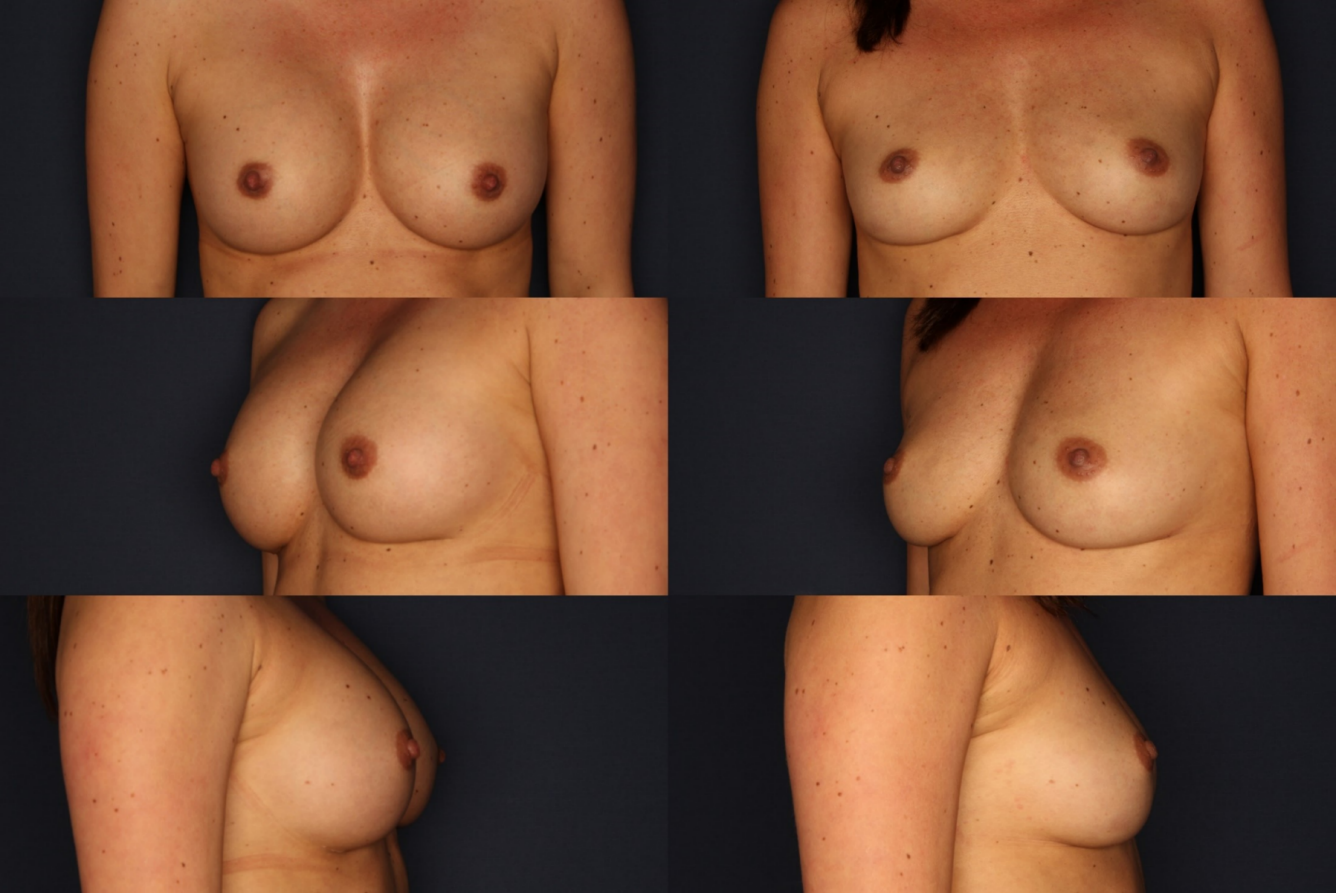
Patient with capsular contracture and symptoms of Breast Implant Illness before implant removal, implant size 260 cc on both sides. On the right, the same patient is depicted after implant removal and immediate reconstruction with autologous fat using the Beauli™ method. Autologous fat was transplanted twice on both sides: 290 cc each side in the first session, and three months later, 250 cc each side in a second session.

In the free-text field, patients had the opportunity to express their medical history beyond the limited scale of the answers. Figure 4 (Fig. 4) exemplifies a patient before and after the operation.

## Results

The results of the survey are explained in Figure 1 (Fig. 1), Figure 2 (Fig. 2), and Figure 3 (Fig. 3), with an exemplary postoperative outcome depicted in Figure 4 (Fig. 4).

Figure 1 (Fig. 1) documents the duration of wearing implants in the patient collective since the initial implantation.

Figure 2 (Fig. 2) presents a comparison of eight symptoms in their intensity before (in red) and after (in blue) implant removal. The results demonstrate a significant reduction in symptoms for all queried individual complaints. Statistical analysis and testing are not applicable due to the scoring system. The graphical representation solely visualizes the development of symptoms in the before-and-after comparison.

It becomes evident that symptoms of the breast implant illness complex can occur at any time after implantation. In our patient collective, the majority of patients who wore implants for a period of five years experienced symptoms.

Figure 3 (Fig. 3) compares the occurrence of comorbidities before (in red) and after (in blue) implant removal. Allergies and susceptibility to infections show a greater decrease after implant removal.

Figure 4 (Fig. 4) depicts on the left a patient with capsular contracture and symptoms of breast implant illness before implant removal, with an implant size of 260 cc on both sides. On the right, the same patient is shown after implant removal and immediate reconstruction with autologous fat using the Beauli™ method. Autologous fat was transplanted twice on both sides: 290 cc each side in the first session, and three months later, 250 cc each side in a second session.

## Discussion

The results of the current survey provide a snapshot of subjective expressions within the treated patient population over the past years (2016 to 2024). With a questionnaire response rate of 45.8%, a representative outcome can be assumed.

This survey does not serve to answer the question of whether a cluster of symptoms, also known as breast implant illness, is significantly improved or completely disappears in the long term after implant removal. However, it can support previously posited assumptions.

It can reinforce the prevailing theory of the pathophysiology of breast implant illness, suggesting that the immune system exhibits an overreaction induced by the amount of silicone in the body [12], [13], [14]. This overreaction can manifest as symptom clusters or as a condition within the autoimmune syndrome induced by adjuvants (ASIA). With the removal of the implant as the trigger for the immune system, the disruptive agent is eliminated, resulting in a reduced or no reaction. Consequently, the symptoms are expected to decrease.

It is noteworthy that there is a clear reduction in the level of complaints, with some symptoms experiencing a decrease of more than half of the previously experienced intensity. However, a complete reduction to zero is not observed for any of the queried symptoms. It is possible that the symptoms may gradually subside over a much longer period than observed in the current collective.

Differentiation between total capsulectomy and non-total capsulectomy within the patient population was not performed. A further study involving two distinct patient groups is needed to determine the influence of the extent of capsule removal [15].

The influence of psychological elements combined with the effects of social media on the mindset of the patients must be considered in evaluating the results. It is conceivable that affected patients, even after implant removal, continue to live in constant fear that migrating silicone could cause further harm. This ongoing preoccupation with the topic may, under certain circumstances, prevent complete remission of the symptoms [16], [17].

A multifactorial approach in the exploration of breast implant illness (BII), incorporating clinical, immunological, and psychological aspects, could fill gaps in the current understanding of BII’s etiology [18]. Future efforts could focus on the interplay between psychological, immunological, and other factors.

Further studies with a larger number of patients are necessary. The lack of a unified definition for the condition complicates the interpretation of existing studies on this symptom complex [19].

The current survey aims to gather information from the perspective of patients regarding changes in symptoms. It can contribute valuable insights into understanding the phenomenon of breast implant illness, allowing the affected individuals to voice their experiences.

The distress experienced by the patients was evident in the free-text field through original statements. Notably, the texts differed in the before-and-after comparison. Often, patients feel that their complaints are not taken seriously, leaving them feeling isolated and prompting them to seek help independently without professional support. This raises the risk of falling into unreliable hands.

It is the responsibility of treating and advising healthcare professionals to become more familiar with and engage with this symptom complex. Further studies will reveal whether implant removal is indeed the crucial element for symptom improvement.

## Notes

### Competing interests

The authors declare that they have no competing interests.
